# Applying improved ddPCR to reliable quantification of MPXV in clinical settings

**DOI:** 10.1128/spectrum.00018-24

**Published:** 2024-05-17

**Authors:** Chudan Liang, Huiqin Yang, Xiaofeng Yang, Zhenyu Long, Yuandong Zhou, Jian Wang, Linjin Fan, Mou Zeng, Yulong Wang, Haipeng Zheng, Zequn Wang, Pengfei Ye, Jingyan Lin, Wendi Shi, Hongxin Huang, Huijun Yan, Jun Qian, Linghua Li, Linna Liu

**Affiliations:** 1 Zhongshan School of Medicine, Sun Yat-sen University, Guangzhou, Guangdong, China; 2 Key Laboratory of Tropical Disease Control (Sun Yat-sen University), Ministry of Education, Guangzhou, Guangdong, China; 3 Institute of Infectious Disease, Guangzhou Eighth People’s Hospital, Guangzhou Medical University, Guangzhou, Guangdong, China; 4 The Third People’s Hospital of Bijie City, Bijie, Guizhou, China; 5 School of Public Health (Shenzhen), Sun Yat-sen University, Shenzhen, Guangdong, China; National Institute of Allergy and Infectious Diseases, Baltimore, Maryland, USA

**Keywords:** monkeypox virus, ddPCR, Mpox, viral loads, quantification

## Abstract

Monkeypox virus (MPXV) poses a global health threat. Droplet digital PCR (ddPCR) holds potential as an accurate diagnostic tool for clinical microbiology. However, there is limited literature on the applicability of ddPCR in clinical settings. In this study, the clinical features of patients with MPXV during the initial outbreak in China in June 2023 were reviewed, and an optimized ddPCR method with dilution and/or inhibitor removal was developed to enhance MPXV detection efficiency. Eighty-two MPXV samples were tested from nine different clinical specimen types, including feces, urine, pharyngeal swabs, anal swabs, saliva, herpes fluid, crust, and semen, and the viral load of each specimen was quantified. A comparative analysis was performed with qPCR to assess sensitivity and specificity and to investigate the characteristics of MPXV infection by analyzing viral loads in different clinical specimens. Consequently, common pharyngeal and gastrointestinal symptoms were observed in patients with MPXV. The optimized ddPCR method demonstrated relatively high sensitivity for MPXV quantification in the clinical materials, with a limit of detection of 0.1 copies/μL. This was particularly evident in low-concentration samples like whole blood, semen, and urine. The optimized ddPCR demonstrated greater detection accuracy compared with normal ddPCR and qPCR, with an area under the curve (AUC) of 0.939. Except for crust samples, viral loads in the specimens gradually decreased as the disease progressed. Virus levels in feces and anal swabs kept a high detection rate at each stage of post-symptom onset, and feces and anal swabs samples may be suitable for clinical diagnosis and continuous monitoring of MPXV.

**IMPORTANCE:**

The ddPCR technique proved to be a sensitive and valuable tool for accurately quantifying MPXV viral loads in various clinical specimen types. The findings provided valuable insights into the necessary pre-treatment protocols for MPXV diagnosis in ddPCR detection and the potentially suitable sample types for collection. Therefore, such results can aid in comprehending the potential characteristics of MPXV infection and the usage of ddPCR in clinical settings.

## INTRODUCTION

Since May 2022, the global spread of mpox disease (formerly known as monkeypox) has become deeply concerning, with 92,783 cases reported as of November 2023 (1). Monkeypox virus (MPXV), the causative agent of mpox, is a double-stranded DNA (dsDNA) virus that can be classified into two distinct clades: clade I and clade II. The current outbreak is caused by the dominant strain closely associated with the B.1 lineages of clade IIb, which is widely geographically widespread and has a faster rate of microevolution (1–4). Interpersonal transmission of MPXV occurs primarily through close contact with body fluids, skin lesions, ulcers, and respiratory droplets of MPXV carriers (1, 5). Mpox is characterized by skin rashes, accompanied by systemic symptoms such as fever, headache, and muscle aches. Lymphadenopathy presents a distinct clinical manifestation that can aid in differentiating mpox from smallpox (6). There are presently no available specific antiviral treatments for MPXV infection; hence, prompt detection is crucial for managing outbreaks and preventing further transmission.

Conventional methods include real-time quantitative polymerase chain reaction (RT-qPCR) and antigen testing, which can be used to confirm MPXV infection (6, 7). However, the accuracy and reliability of the results may be impacted by their sensitivity limitations. A third-generation PCR technology, digital droplet PCR (ddPCR), has emerged as a potentially effective tool for microbiological detection recently (8). The unique capabilities of ddPCR allow for accurate quantification without reliance on Cq values and standard curves (9). ddPCR has been applied to the identification and quantification of several pathogens, such as SARS-CoV-2, human immunodeficiency virus (HIV), human papilloma virus (HPV), and *Mycobacterium tuberculosis* (10–13). Therefore, ddPCR has potential as a diagnostic technique in clinical microbiology. However, there is limited literature on the applicability of ddPCR in clinical settings.

**Fig 1:**
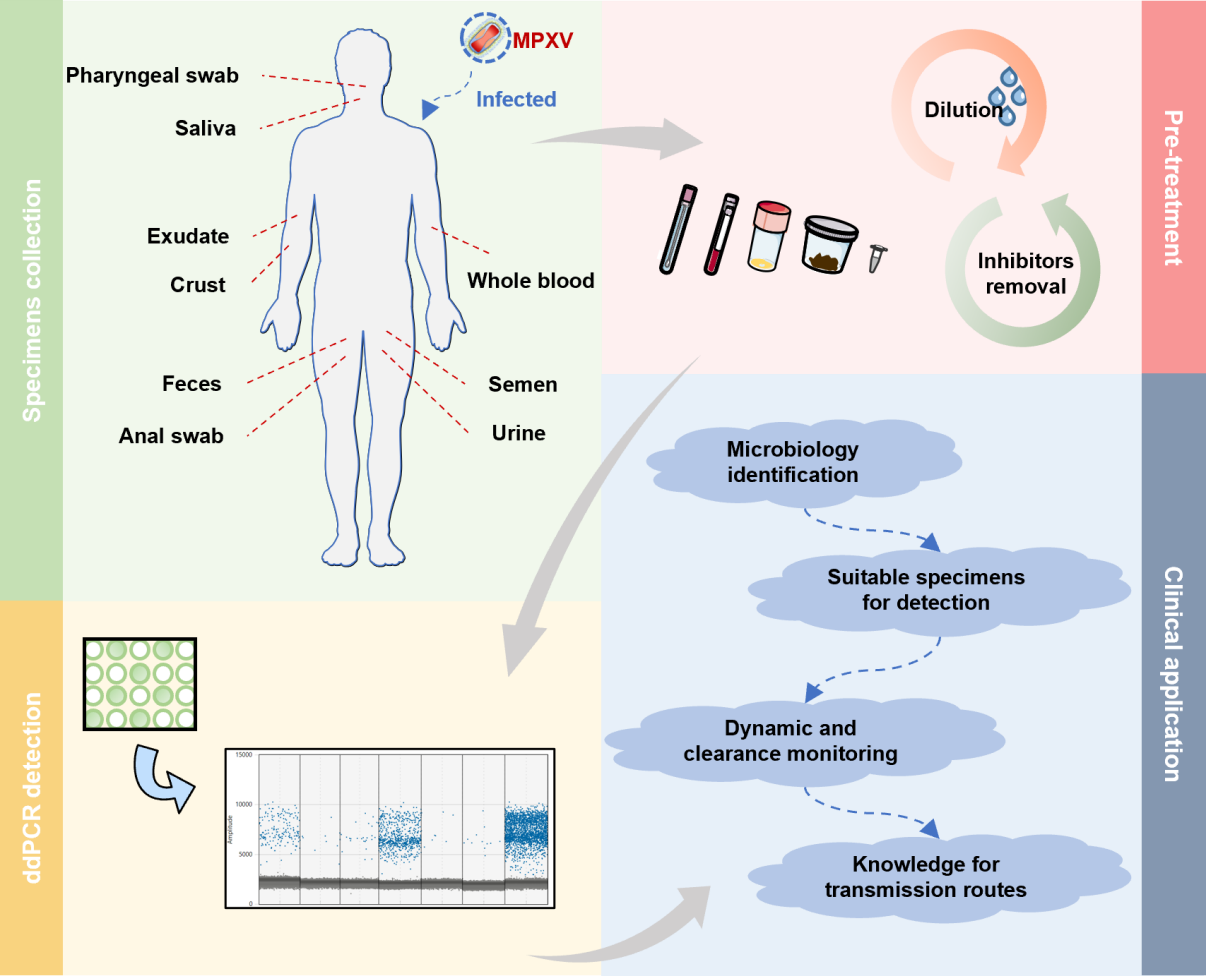
Flowchart of MPXV detection clinically using the optimized ddPCR method. Clinical specimens should be collected based on the patient’s stage of onset. Each sample should be pre-treated accordingly, including dilution and/or inhibitor removal. Once optimized, perform the ddPCR assay to quantify MPXV.

In this study, a reliable and improved ddPCR assay for MPXV was developed through method validation and clinical testing (flowchart shown in Fig. 1). The findings provided valuable insights into the necessary pre-treatment protocols for MPXV diagnosis in ddPCR detection, the potentially suitable sample types for collection, and the potential clinical features of MPXV infection.

## RESULTS

### Pharyngeal and gastrointestinal symptoms are common in patients with MPXV

There has been an outbreak of mpox disease in China since June 2023. An analysis of 17 confirmed cases of MPXV infection in June was undertaken to investigate the clinical features of this infection (Tables 1 and 2). All individuals were adult males under 45 years of age, and none had been vaccinated against smallpox. Seventy-six percent of them had previous infections with sexually transmitted pathogens, such as *Treponema pallidum* (TP) and HIV. Skin eruptions were a prodrome in the majority of the patients. Importantly, in addition to the typical clinical features of skin lesions, each case presented clinical symptoms affecting the pharyngeal and gastrointestinal systems, such as pharyngalgia/pharyngeal congestion, antiadoncus, and diarrhea. Four of the cases also had proctitis or colitis. However, these symptoms represented only a small percentage of the overall global data (1).

**TABLE 1:** Baseline characteristics of confirmed MPXV cases^*b*^

	Participants (*n* = 17)
Sex	
Male	17 (100%)
Age, years	
Median	33 (25–45)
Anti-smallpox vaccination	
Unvaccinated	17 (100%)
Pre-sexually transmitted pathogens	
Infected	13 (76.47%)
*Treponema pallidum*	10 (58.82%)
HIV	7 (41.18%)
Others^*a*^	5 (29.41%)
Co-infected	7 (41.18%)
Uninfected	4 (23.53%)

^*a*^ Includes HSV-2 and HPV.

^*b*^ Data were *n* (%).

**TABLE 2:** Clinical presentation of confirmed MPXV cases^*b*^

	Participants (n = 17)
Prodromes	
Skin lesions	14 (82.35%)
Fever	3 (17.65%)
Clinical features	
Rash	17 (100%)
Fever	11 (64.71%)
Exhaustion	3 (17.65%)
Lymphadenopathy^*a*^	14 (82.35%)
Pharyngalgia/pharyngeal congestion	15 (88.24%)
Antiadoncus	11 (64.71%)
Diarrhea	5 (29.41%)
Proctitis/colitis	4 (23.53%)
Localization of skin lesions	
Body	15 (88.24%)
Face	12 (70.59%)
Genitals	11 (64.71%)
Anal margin	3 (17.65%)
Groin	7 (41.18%)

^*a*^ Includes cervical and inguinal lymph nodes.

^*b*^ Data were *n* (%).

### The optimized ddPCR is conducted to detect MPXV in various types of clinical samples

**Fig 2:**
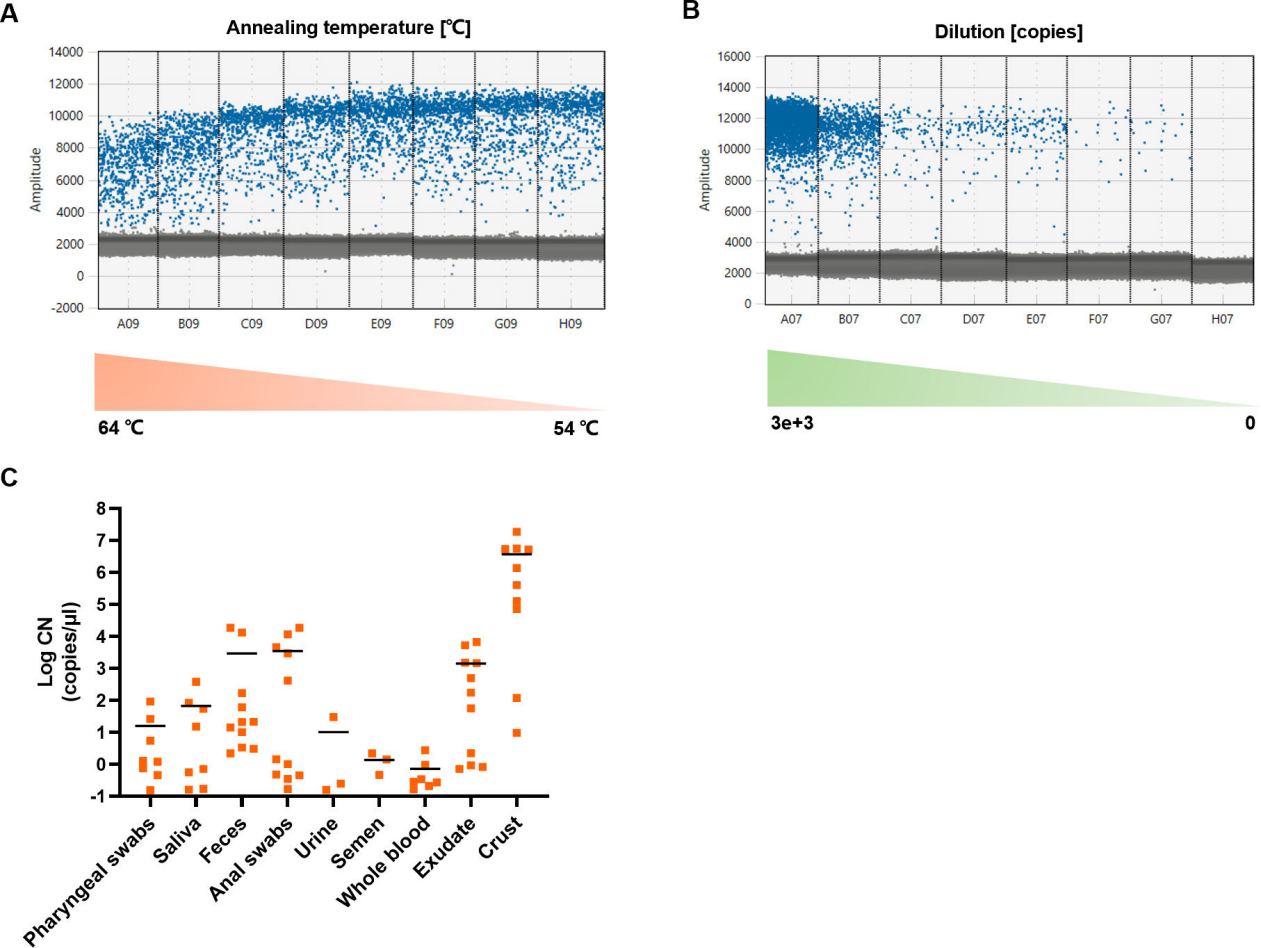
Establishment of the ddPCR method for MPXV targeting F3L. (A) The optimal annealing temperature for ddPCR using a positive standard of F3L DNA fragmentation. (B) The detectable range of ddPCR using a positive standard of F3L DNA fragmentation at a 10-fold dilution. Panels C07–E07 were duplicated samples. (C) The copy numbers of MPXV in each clinical sample were calculated using the optimized ddPCR assay. Each dot represents one sample, and the data are shown with the mean.

To determine whether the characteristics of their viral loads were different, a sensitive detection method was indeed used. We collected various clinical sample types, including pharyngeal swabs, saliva, feces, anal swabs, urine, semen, whole blood, exudate, and crust. Therefore, we performed an optimized ddPCR assay targeting the F3L gene. Initially, the method was constructed, and its applicability was validated using experimental samples. The experimental observations revealed that positive droplets could be effectively distinguished from negative droplets with an optimal annealing temperature of 58°C (Fig. 2A). The ddPCR assay exhibited exceptional sensitivity and reproducibility when using continuously diluting MPXV F3L DNA fragment samples. The limit of detection (LOD) was as low as 0.1 copies/μL, with a coefficient of variation (CV) of less than 10% (Fig. 2B). The optimized assay did not cross-react with related viruses, such as cowpox virus (CPXV), variola virus (VARV), varicella-zoster virus (VZV), human herpesvirus type 1 (HHV-1), and human herpesvirus type 2 (HHV-2). Notably, the no template control (NTC) showed no quantification signals. After validating the methodology, we extended our study to quantify 82 clinical MPXV samples (Fig. 2C). The results demonstrated that the optimized ddPCR assay could be used to accurately quantify MPXV in diverse clinical samples.

### The optimized ddPCR method increases the sensitivity of clinical MPXV detection compared with non-optimized ddPCR

**Fig 3:**
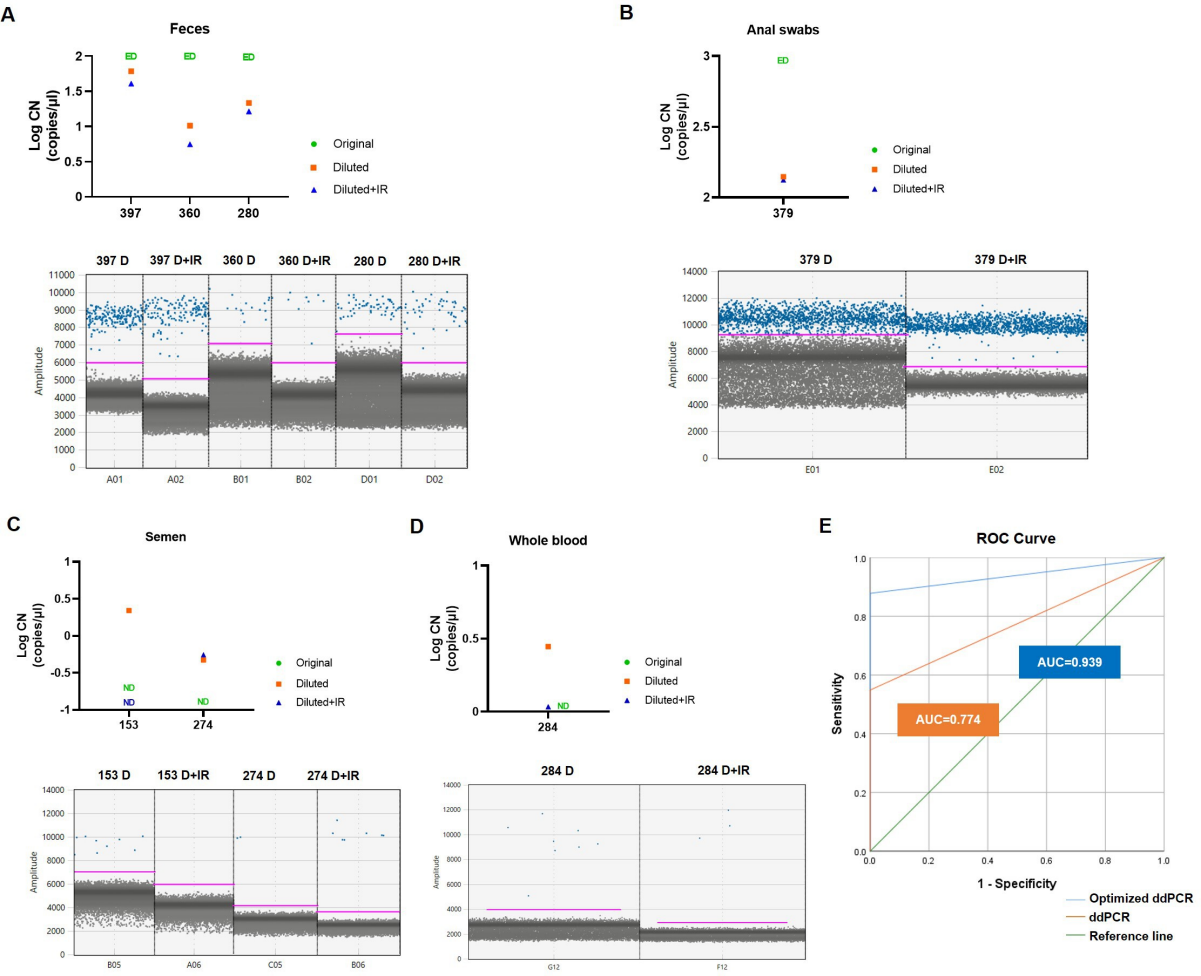
Effectiveness of the ddPCR assay for MPXV after optimization. (A–D) The viral copy numbers in feces, anal swabs, semen, and whole blood samples. ED, exceed detection limit; ND, not detected; D, dilution; IR, inhibitors removal. (E) The receiver operating characteristic (ROC) curve comparing ddPCR and optimized ddPCR. The value of AUC is shown in the figure.

The optimization procedure for clinical samples involved dilution and removal of inhibitors. Despite the purification of the sample during nucleic acid extraction, we encountered discordant results between ddPCR and qPCR experiments prior to optimization. This inconsistency could be due to impurities in the clinical samples that hindered the PCR reactions (14, 15). The results of the experiment showed that dilution and inhibitor removal led to a clearer differentiation between positive and negative droplets (Fig. 3A and B). After optimization, the ddPCR assay significantly reduced background noise. The minimum amplitude was reached at approximately 2,000 (Fig. 3A through D). For sample types with relatively low MPXV viral loads, such as semen and whole blood, these improved measures allowed accurate measurements. Specifically, the concentrations of samples 153, 274, and 284, which previously showed discrepancies in quantification between ddPCR and qPCR prior to sample optimization, were determined to be 2.196 copies/μL, 0.471 copies/μL, and 2.788 copies/μL, respectively (Fig. 3C and D). These samples also exhibited result instability in repeated experiments before optimization. The optimized ddPCR exhibited increased sensitivity and improved detection accuracy, as evidenced by an area under the curve (AUC) of 0.939, compared with the non-optimized ddPCR with an AUC of 0.774 (Fig. 3E). These positive findings emphasized the importance of pre-treating clinical samples prior to detection. The optimized ddPCR technique was shown to be highly effective in enhancing detection sensitivity.

### The optimized ddPCR method demonstrates superior sensitivity in clinical performance compared with optimized qPCR

**Fig 4:**
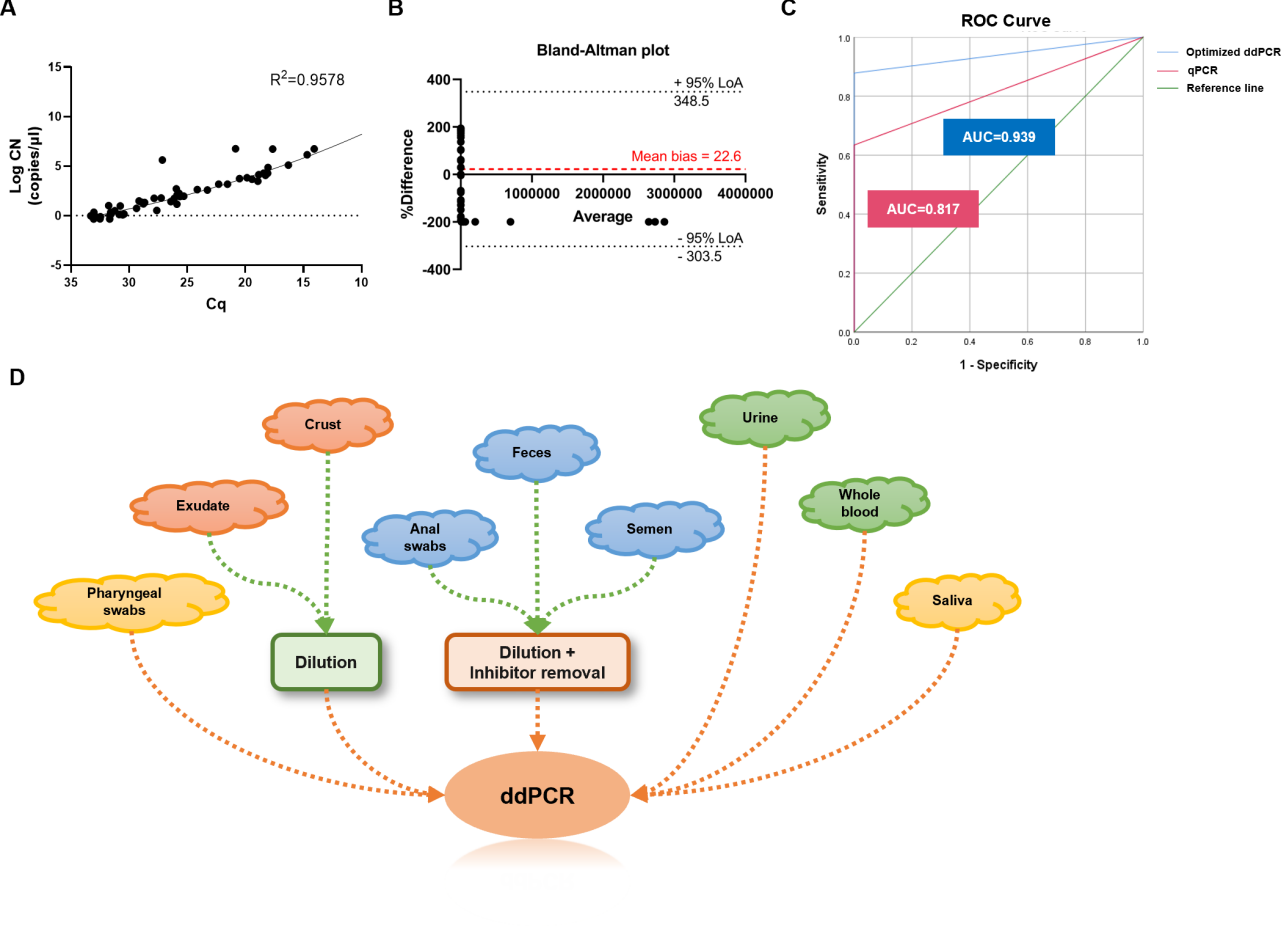
Comparative analysis between optimized ddPCR and qPCR for MPXV and collection strategies of clinical specimen types for ddPCR detection clinically. (A) Correlation between optimized ddPCR and qPCR assays. The Cq values measured by qPCR in each clinical sample with the same pre-treatment are represented on x-axis, whereas their corresponding copy numbers evaluated by ddPCR are represented on the y-axis. (B) Bland-Altman plots for quantifying MPXV clinical samples using optimized ddPCR and qPCR methods. The red line represents the average of measures, whereas the black line represents the 95% limits of agreement. (C) The receiver operating characteristic (ROC) curve comparing ddPCR and qPCR in clinical settings. The value of AUC area is shown in the figure. (D) Appropriate pre-treatment strategies employed for various clinical specimen types when using ddPCR for detection.

In order to assess the optimized ddPCR method’s clinical performance, a comparison was made between the optimized ddPCR and qPCR. The two methods exhibited an overall percent agreement of 78.05% with the detection results for clinical samples. However, this varied across different specimen types (Table 3). Higher detection percent agreement was observed in samples of crust (100.00%), pharyngeal swabs (90.91%), feces (90.91%), anal swabs (81.82%), saliva (81.82%), and exudate (81.82%). High MPXV viral loads were found in these samples (Fig. 2C). An analysis of correlation and agreement between the two methods was performed. The data indicated a moderate goodness of fit, with an R^2^ value of 0.9578 and Sy.x of 0.3841 (Fig. 4A). The Bland-Altman analysis showed an acceptable agreement between the optimized ddPCR and qPCR methods, with *P* value > 0.05 in paired *t*-test (Fig. 4B).

**TABLE 3:** The consistency results between qPCR and ddPCR

Sample types	ddPCR	qPCR		Agreement
		Positive	Negative	
Pharyngeal swab	Positive	7	1	90.91%
	Negative	0	3	
Saliva	Positive	6	2	81.82%
	Negative	0	3	
Feces	Positive	10	1	90.91%
	Negative	0	0	
Anal swab	Positive	9	2	81.82%
	Negative	0	0	
Urine	Positive	1	2	71.43%
	Negative	0	4	
Semen	Positive	1	2	33.33%
	Negative	0	0	
Whole blood	Positive	0	7	0.00%
	Negative	0	0	
Exudate	Positive	9	2	81.82%
	Negative	0	0	
Crust	Positive	10	0	100.00%
	Negative	0	0	
Total	Positive	53	18	78.05%
	Negative	0	11	

Furthermore, the detection sensitivities for all clinical samples were 64.63% and 86.59% for qPCR and ddPCR, respectively. Upon analyzing individual sample types, ddPCR demonstrated superior sensitivity to qPCR across all nine specimen types (Table 4). This was particularly evident in samples with low virus loads such as urine (42.86% in ddPCR vs. 14.29% in qPCR), whole blood (100.00% vs. 0.00%), and semen (100.00% vs. 33.33%). It is worth highlighting that both qPCR and ddPCR assays exhibited a specificity of 100.00%, and no quantification was detected in the clinical samples collected from healthy volunteers (Table 4). The optimized ddPCR method demonstrated superior detection accuracy when compared with optimized qPCR (Fig. 4C). These findings indicated that the optimized ddPCR method presents exceptional sensitivity and specificity in quantifying MPXV in nine different types of clinical specimens, outperforming optimized qPCR in terms of sensitivity.

**TABLE 4:** The sensitivity and specificity results for MPXV in different clinical samples by qPCR and ddPCR

Sample types	qPCR		ddPCR	
	Sensitivity [95% CI]	Specificity [95% CI]	Sensitivity [95% CI]	Specificity [95% CI]
Pharyngeal swabs	63.64% [30.79% to 89.07%]	100.00% [29.24% to 100.00%]	72.73% [39.03% to 93.98%]	100.00% [29.24% to 100.00%]
Saliva	54.55% [23.28% to 83.25%]	100.00% [29.24% to 100.00%]	72.73% [39.03% to 93.98%]	100.00% [29.24% to 100.00%]
Feces	90.91% [58.72% to 99.77%]	100.00% [29.24% to 100.00%]	100.00% [71.51% to 100.00%]	100.00% [29.24% to 100.00%]
Anal swabs	81.82% [48.22% to 97.72%]	100.00% [29.24% to 100.00%]	100.00% [71.51% to 100.00%]	100.00% [29.24% to 100.00%]
Urine	14.29% [0.36% to 57.87%]	100.00% [29.24% to 100.00%]	42.86% [9.90% to 81.59%]	100.00% [29.24% to 100.00%]
Semen	33.33% [0.84% to 90.57%]	/	100.00% [29.24% to 100.00%]	/
Whole blood	0.00% [0.00% to 40.96%]	100.00% [29.24% to 100.00%]	100.00% [59.04% to 100.00%]	100.00% [29.24% to 100.00%]
Exudate	81.82% [48.22% to 97.72%]	100.00% [29.24% to 100.00%]	100.00% [71.51% to 100.00%]	100.00% [29.24% to 100.00%]
Crust	100.00% [69.15% to 100.00%]	/	100.00% [69.15% to 100.00%]	/
Total	64.63% [53.30% to 74.88%]	100.00% [83.89% to 100.00%]	86.59% [77.26% to 93.11%]	100.00% [83.89% to 100.00%]

### Pre-treatment strategies should be tailored to the clinical specimen in ddPCR detection

Based on the findings mentioned above, different pre-treatment methods should be used for various clinical specimens in ddPCR detection for clinical practice. Upper respiratory tract specimens, such as pharyngeal swabs and saliva, can be detected with or without improved treatments. Skin and alimentary tract samples, including exudate, crust, feces, and anal swabs, require dilution and removal of inhibitors to mitigate impurities before ddPCR assay. To guarantee accurate results, it is crucial to properly dilute and treat semen samples from the genital tract to eliminate inhibitors before detection. Additionally, for precise quantification of specimens with low viral loads, such as urine and whole blood collected from the urinary tract and bloodstream, it is recommended to use ddPCR instead of qPCR (Fig. 4C).

### Skin lesion samples show a rise in MPXV viral loads during the later stage after symptom onset

**Fig 5:**
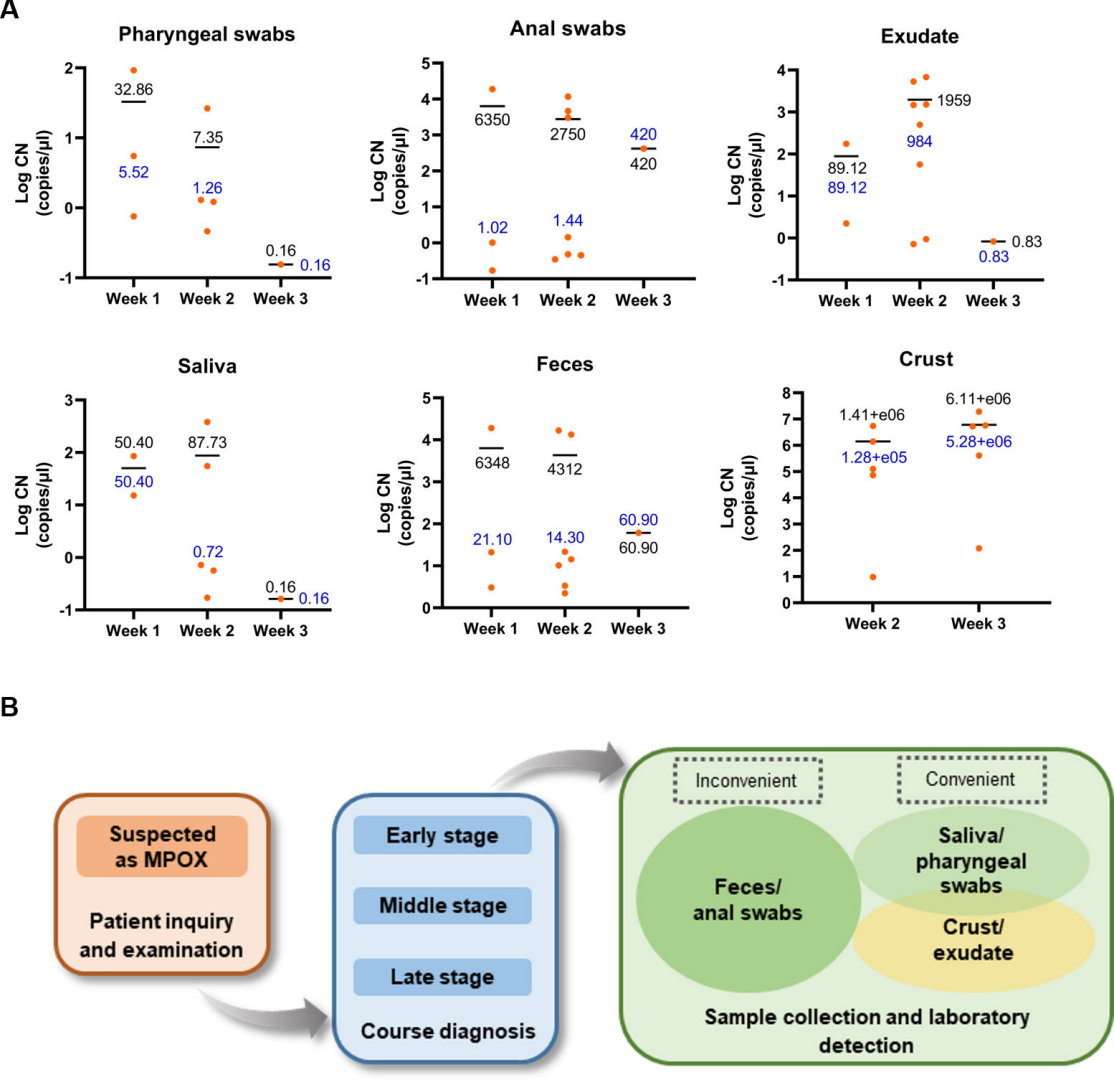
Viral loads of MPXV in various clinical specimens at different onset times and collection strategies of clinical specimen types for ddPCR detection. (A) The changes in viral loads of MPXV in various clinical sample types as the onset progressed. The copy numbers were measured by the optimized ddPCR assay. Each dot represented one sample, and the text indicated the copy numbers of samples in non-logarithmic value. The black text represented the mean copy number, whereas the blue text represented the median. (B) Flowchart for obtaining appropriate clinical sample types from individuals suspected of MPXV infection at various stages of onset. When selecting sample types for detection, consider both the detectability and convenience of samples, taking into account the patient’s onset.

The optimized ddPCR was utilized to ascertain the abundance of MPXV nucleic acid in each specimen. The viral loads in crust specimens were the highest, followed by anal swabs, exudate, and pharyngeal swabs, all of which were pre-treated in the same manner, when detecting in the same volume of matrix solution (Fig. 2C; Fig. S1A). Similarly, when detecting in the same volume of biomaterials, saliva samples exhibited a higher viral load than urine, semen, and whole blood (Fig. 2C). In MPXV infection, viral loads were more abundant and easily detected in the specimens from the skin, upper respiratory tract, and alimentary tract of patients who experience pharyngeal and gastrointestinal distress symptoms. A comparison was conducted to evaluate the changing of MPXV viral loads in different sample types during disease progression. As a whole, viral loads gradually declined as mpox progressed, eventually reaching very low levels by the third week after symptom onset. However, the viral levels of crust continued to increase over time (Fig. 5A). Skin lesions from the later stage of the disease may have a high level of infectivity, which can be used to monitor the clearance of MPXV.

### Different clinical samples may be collected at various stages of post-symptom onset for MPXV diagnosis

When investigating the characteristics of MPXV infection using the optimized ddPCR method, high concentrations of MPXV were observed in the alimentary tract specimens, including feces and anal swabs (Fig. 2C and 5A; Fig. S2C), and remained consistently high throughout the entire onset phase within the same individual (Fig. S1B). In addition, the optimized ddPCR detection produced negative results for pharyngeal swabs in two patients with MPXV infection, whereas their feces or anal swab samples remained positive during the later stage of the disease. Given the challenges of obtaining crust and exudate specimens during the initial phase of onset, and their limited usefulness due to their non-invasive nature, feces and anal swabs may be more suitable specimen types for identifying MPXV clinically and monitoring virus changes. When diagnosing MPXV, different clinical specimens may be collected for testing depending on the patient’s stage of onset, considering detectability and convenience (Fig. 5B).

## DISCUSSION

Since mpox broke out on a small scale in China in June 2023, the observation of common pharyngeal and gastrointestinal symptoms in patients with MPXV has led us to focus on the virus’s abundance in different sites and tissue tropism. We examined the viral loads in specimens taken from the upper respiratory and alimentary tracts, including pharyngeal swabs, saliva, anal swabs, and feces, which showed a high concentration of the virus. Previous studies have reported high viral loads in skin lesions and anal samples (16, 17). In this study, we attempted to carry out experiments based on symptoms and viral loads. Although the World Health Organization (WHO) recommends using skin lesion samples for detecting MPXV (18), our results demonstrated that using the optimized ddPCR, specimens collected from the alimentary tract, such as feces and anal swabs, consistently displayed high viral loads throughout the course of the disease. Consequently, feces and anal swabs may be more effective specimen types for clinical detection and quantification of MPXV. If the required specimens are unavailable, alternative specimens such as saliva and lesion *in situ* samples should also be taken into account (19, 20).

Specimens from the alimentary tract, such as feces and anal swabs, were found to contain more inhibitors, which can affect the effectiveness of ddPCR. Although ddPCR has been shown to offer advantages over qPCR in terms of enhanced amplification efficiency (21, 22) and reduced impact from inhibitors present in samples (8, 15, 23), it should be noted that inhibition can also influence the fluorescence amplitude in ddPCR detection (24). After developing a ddPCR method for MPXV quantification, we further improved its accuracy and reliability by diluting and/or removing inhibitors. This highlights the significance of pre-treatment of clinical specimens prior to detection, which can expand the application of ddPCR in identifying microorganisms in clinical microbiology practice.

Biases can occur at different stages of research: when reading up on the field, specifying and selecting the study sample, executing the experimental maneuver, measuring exposures/outcomes, analyzing data, interpreting results, and publication (25). These biases may have been introduced due to a lack of internal validity (25, 26). To minimize bias, it is important to document the process, establish standard values, monitor procedures, and make necessary adjustments during the experiment. We implemented measures to control accuracy of the results during the experiments. The operator conducting the experiments has extensive experience, and we followed strict specifications and manufacturer’s instructions. Each experiment was performed by the same person using identical reagent consumables throughout. The samples underwent complete lysis, and the quality of the DNA was tested and controlled.

Some indirect evidence suggests an increased risk of fecal-oral transmission of MPXV, for instance, high viral loads were quantified in the alimentary tract and upper respiratory specimens. Infectious virus particles were also discovered in stool samples (27), indicating the possibility of viral shedding from the salivary glands (SGs) and gastrointestinal tract. Nevertheless, these findings are not sufficient to directly support the hypothesis. Building on other previous confirmatory research (28–30) and to advance our research, our next project entails investigating the replication and propagation of the virus in the salivary glands and gastrointestinal tracts, observing corresponding inflammation and tissue injury and isolating live virus from oropharyngeal and fecal samples. There are certain limitations to our study, such as the absence of MPXV clinical samples from the incubation period and convalescence. These samples would further confirm the optimum specimen types for early diagnosis and monitoring of viral excretion and clearance, which are imperative for pandemic control.

In conclusion, we have successfully developed an optimized ddPCR method to accurately identify and sensitively quantify MPXV. We emphasize the importance of pre-treating samples prior to detection and have investigated potential alternative sample types for clinical detection using the optimized ddPCR method. This enhances our understanding of ddPCR in clinical settings and the transmission properties of MPXV. Such comprehension is crucial in pandemic management and the development of effective preventive measures for mpox.

## MATERIALS AND METHODS

### Patients and data collection

Our study included 17 patients who were diagnosed with mpox by PCR test with Skin swab or fluid from blisters and were admitted to Guangzhou Eighth People’s Hospital in June 2023. The diagnostic and discharge criteria for MPXV adhere to the guidelines outlined in the “ [(31) Edition]” released by China. The patients’ clinical data including laboratory data were collected from medical records. Other clinical and tracking information was obtained directly from the patients through a survey. Additionally, the study was conducted with the approval of the Ethics Committee of Guangzhou Eighth People’s Hospital, Guangzhou Medical University.

### Clinical specimens

A total of 103 clinical specimens were gathered from six different body parts, comprising nine different types, including 82 MPXV samples (Table 5). Twenty-one clinical specimens were collected from healthy volunteers to serve as controls, including pharyngeal swabs (*n* = 3), saliva (*n* = 3), feces (*n* = 3), anal swabs (*n* = 3), urine (*n* = 3), whole blood (*n* = 3), and exudate (*n* = 3). These specimens were collected from 17 confirmed cases of mpox during the outpatient examinations and hospitalizations following its initial outbreak in Guangzhou, China in June 2023, and they were subsequently used for experiments. The dry swabs were stored in a viral transport medium (VTM), and the biomaterials were preserved with the addition of penicillin and streptomycin.

**TABLE 5:** Clinical specimen types and number of MPXV

Locations	Specimen types	Number
Upper respiratory tract	Pharyngeal swabs	11
	Saliva	11
Alimentary tract	Feces	11
	Anal swabs	11
Urinary tract	Urine	7
Genital tract	Semen	3
Blood	Whole blood	7
Skin	Exudate	11
	Crust	10

### DNA extraction

The QIAamp DNA mini kit (Qiagen) was utilized to extract DNA. Briefly, 200 µL of VTM from each sample like pharyngeal swabs, anal swabs, exudate, and crust, 200 µL of matrix from each biomaterial such as saliva, urine, semen, and whole blood, as well as 200 mg of feces samples were processed for DNA extraction in accordance with the manufacturer’s instructions. The resulting DNA was then eluted in 50 µL DNase-free water, and their concentrations were measured along with the A260/A280 and A260/A230 ratios. The A260/A280 ratio of the DNA was between 1.7 and 2.0, and the A260/A230 ratio was between 2.0 and 2.2. The resulting DNA samples were stored at −80°C.

### Droplet digital PCR

The ddPCR assay was performed using the QX200 (BIO-RAD). The F3L gene was targeted with the forward primer, 5′-CGGGATACCCGCTAACAATAAA-3′, and the reverse primer, 5′-GATGCCACTGCTGGATTACA-3′. The reaction mixture, consisting of 2× QX200 ddPCR EvaGreen Supermix, forward and reverse primers (100 nM each), DNA template, and DNase/RNase-free water, was carefully put together to a final volume of 20 µL. The emulsified samples were generated using the QX200 droplet generator. Thermal cycling was performed according to the following protocol: enzyme activation at 95°C for 5 minutes, denaturation at 95°C for 30 seconds, annealing/extension at 58°C for 1 minute for 40 cycles, and signal stabilization at 4°C and 90°C for 5 minutes each, followed by an infinite 4°C hold. Resultant data were collected using the QX200 reader and analyzed using QX Manager 2.1 Standard Edition Software.

### Quantitative PCR

The QuantStudio five real-time PCR system (Applied Biosystems) was used for conventional qPCR assay. The reaction mix, containing 2× MagicSYBR Mixture, the forward and reverse primer (used in the ddPCR assay above, 1 µM each), DNA template, and DNase/RNase-free water was built up to a final volume of 20 µL. The cycling protocol was as follows: pre-denaturation at 95°C for 30 seconds, denaturation at 95°C for 5 seconds, annealing/extension at 60°C for 30 seconds for 40 cycles, and, finally, melting curve analysis, followed by an infinite 4°C hold. The experimental data were analyzed using QuantStudio Design and Analysis software. The qPCR validation experiments for MPXV quantification were conducted (Fig. S2), and the copy numbers of the samples were evaluated using the standard equation.

### Inhibitor removal

A one-step PCR inhibitors removal procedure was performed using a kit (ZYMO RESEARCH), according to the manufacturer’s instructions. The recycled DNA was then stored at −80°C.

### LOD analysis

The LOD for MPXV was determined by a positive standard of F3L DNA fragmentation continuously at a 10-fold dilution. The F3L DNA fragment was amplified from MPXV. A negative control (NTC) was also included.

### Correlation and agreement analysis

The correlation and agreement of methods were analyzed using GraphPad Prism (version 9) software. Nonlinear regression analysis was used to assess goodness of fit. Both R-squared (R^2^) and standard deviation of residuals (S) were used to assess correlation between methods (32). A higher R^2^ indicates a better fit of the model to the data, whereas a smaller S value indicates closer predictions of the data values. In GraphPad Prism, the S value is written as Sy.x. Additionally, Bland-Altman analysis (33) and paired *t*-test were conducted to compare the agreement of methods. The difference in values was converted to (100*(A−B)/average)% for analysis. The mean bias and 95% limit of agreement (LOA) were evaluated, and the plots should fall within the range of LOA. In the paired *t*-test, *P* > 0.05 indicates no significant difference between the two methods.

### Receiver operating characteristic analysis

Clinical sensitivity is defined as the percentage of true positives among all patients with a disease, whereas clinical specificity is the percentage of true negative tests among all persons without the disease (34). The study analyzed these parameters by using two-way contingency analysis, and the MedCalc was used for the receiver operating characteristic (ROC) curve analysis. The proximity of the ROC curve to the upper left corner indicates a higher overall test accuracy. The AUC is the parameter that measures the ability to differentiate between two diagnostic groups (diseased/normal).

### Statistical analysis

Statistical analyses were performed using GraphPad Prism (version 9) and MedCalc software. The statistical tests and criteria for evaluation were explained in detail above.

## SUPPLEMENTAL MATERIAL

Supplemental figures spectrum.00018-24-s0001.docx Fig. S1 and S2.

Supplemental material spectrum.00018-24-s0002.pdf Ethical approval.

Supplemental tables spectrum.00018-24-s0003.xlsx Table S1 and S2.

ASM does not own the copyrights to Supplemental Material that may be linked to, or accessed through, an article. The authors have granted ASM a non-exclusive, world-wide license to publish the Supplemental Material files. Please contact the corresponding author directly for reuse.
