# Supplementary material for: Applying improved ddPCR to reliable quantification of MPXV in clinical settings
Source: Microbiol Spectr. 2024 May 17;12(7):e00018-24. doi: 10.1128/spectrum.00018-24 (PMC11218477; doi:10.1128/spectrum.00018-24)
Supplement: Supplemental figures — Fig. S1 and S2. [file spectrum.00018-24-s0001.docx]

**Supplement figures and legends**


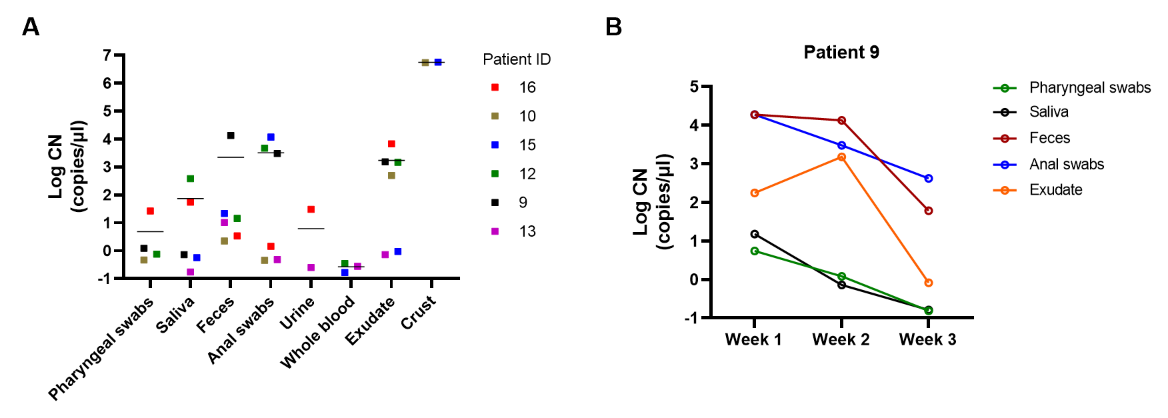


**Fig. S1 Viral loads of MPXV in various clinical samples from multiple individuals at the same stage of onset and the changes of MPXV concentrations over time in the same individual.** (A) Viral loads of MPXV in various clinical samples at the second week of post-symptom onset. The optimized ddPCR method was used to measure the copy numbers of each sample. (B) The changes in viral loads of MPXV in various clinical sample types over time within the same individual. The copy numbers were measured using the optimized ddPCR assay.


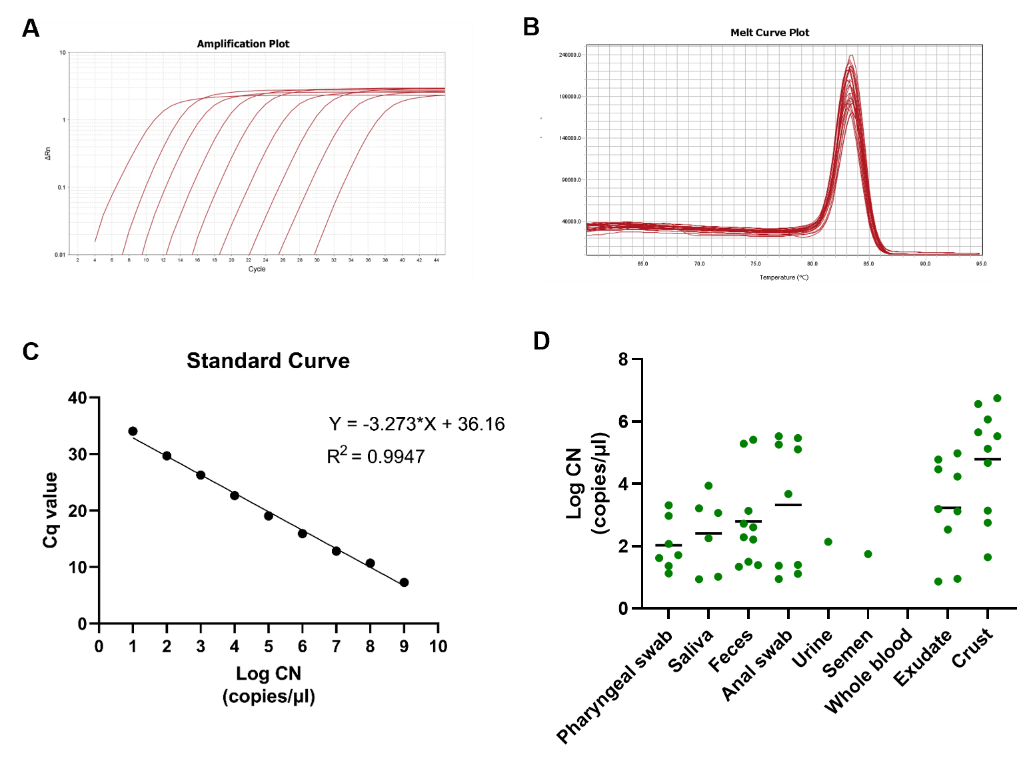


**Fig. S2 Establishment of qPCR method for MPXV quantification.** (A - B) The amplification cure and melting cure of MPXV F3L using the qPCR method. A ten-fold dilution standard was used for the experiments. (C) The standard curve of MPXV using the ten-fold dilution standard of F3L in the qPCR assay. The equation and the correlation coefficient R^2^ were included in the figure. (D) The copy numbers of MPXV in each clinical sample evaluated by qPCR. The data was showed with the mean.
