# Supplementary material for: Applying improved ddPCR to reliable quantification of MPXV in clinical settings
Source: Microbiol Spectr. 2024 May 17;12(7):e00018-24. doi: 10.1128/spectrum.00018-24 (PMC11218477; doi:10.1128/spectrum.00018-24)
Supplement: Supplemental material — Ethical approval. [file spectrum.00018-24-s0002.pdf]

初审

广州医科大学附属市八医院医学伦理委员会

编号：AF/SC-01/01.1

伦理审查意见函

市八伦字号 科 202311248

项目名称	猴痘的临床与基础研究		
项目类别	临床科研项目		
组长单位 / 主要研究者	NA		
本院项目负责人 / 职称	李凌华 /主任医师	研究申办者：NA	
审查日期	2023 年 06 月 28 日	审查方式：☐会议审查 ☑快速审查	
会议地点	NA		
审查委员	何凯茵、李丹		
审查类别	初始审查		
递交文件目录	1、伦理审查申请表；<br>2、研究方案【版本号:GZ8HHDYJ-V1/版本日期:2023-06-13】；<br>3、知情同意书【版本号:GZ8HHDYJZQTYS-V1/版本日期:2023-06-16】；<br>4、主要研究者专业履历；<br>5、研究经济利益冲突声明；<br>6、研究人员名单及职责分工；<br>7、研究材料诚信承诺书。		
审查结果及意见	审查结果：<br>☐同意 ☑必要的修改后同意 ☐不同意 ☐终止或者暂停已同意的研究<br><br>审查意见：建议对知情同意书进行如下修改：<br>1、“二、风险与不适”中采血与采集咽拭子属于临床常规诊疗，其产生的风险不属于该研究的风险，建议删除；<br>2、增加关于“补偿”的说明；<br>3、“六、个人信息的保密”中第 5 点“这项研究结果发表时，将不会披露您个人的任何资料”表述有误，应修改为“将不会披露任何可能识别您个人身份的资料”；<br>4、补充告知受试者将获得签字的知情同意书副本。<br><br>跟踪审查频率：12 个月<br>本文件有效期： NA 失效日期：NA<br><br>主任委员（或副主任委员）签字：<br>广州医科大学附属市八医院医学伦理委员会<br>日期：2023 年 6 月 29 日		

广州医科大学附属市八医院医学伦理委员会

注意事项：

1. 本项临床试验/研究应当在伦理委员会同意进行之日起 1 年内实施，逾期未实施的，本文件自行废止。
2. 自同意研究之日起，应在批准的跟踪审查频率到期前至少一个月以研究进展报告的形式递交年度/定期跟踪审查申请。
3. 审查意见为“必要的修改后同意”的，请在文件签发之日起一个月内递交复审申请，逾期不递交的伦理委员会有权不予受理。
4. 审查意见为“终止或暂停已同意的研究”、“不同意”的，可在文件签发之日起 2 周内以书面形式向伦理委员会就有关事项做出解释或提出申诉，逾期将不再予以受理。
5. 凡涉及人类遗传资源出口或者按照国家规定必须经有关部门专项审批的研究内容，均须遵循国家相关法规及规定获得有关部门批准，并及时将批准文件交伦理委员会备案后方可实施。
6. 研究过程中，对研究方案、知情同意书、招募广告、研究者手册等所作的任何修改，都应及时以修正案审查申请的形式提交伦理委员会进行审查；
7. 本中心发生的可疑且非预期严重不良反应，应当及时向伦理委员会报告；
8. 本中心发生的重要的方案违背和重要的不依从相关法规事件，应及时提交“不依从/违背方案报告”；
9. 研究过程中，增加受试者风险或者显著影响临床试验实施的改变以及任何可能对受试者的安全或者临床试验的实施产生不利影响的新信息都应及时向伦理委员会报告。
10. 经伦理委员会批准的涉及人的生物医学研究项目在实施前，研究项目负责人应当按照国家相关规定在医学研究登记备案信息系统进行登记。

申明：本伦理委员会的组成及工作程序符合 GCP 原则及国家相关管理规定。

伦理委员会地址：广州市白云区华英路 8 号　联系人：周蓉　联系电话：020-37436408
